# Providing Care Beyond Therapy Sessions With a Natural Language Processing–Based Recommender System That Identifies Cancer Patients Who Experience Psychosocial Challenges and Provides Self-care Support: Pilot Study

**DOI:** 10.2196/35893

**Published:** 2022-07-29

**Authors:** Yvonne W Leung, Bomi Park, Rachel Heo, Achini Adikari, Suja Chackochan, Jiahui Wong, Elyse Alie, Mathew Gancarz, Martyna Kacala, Graeme Hirst, Daswin de Silva, Leon French, Jacqueline Bender, Faye Mishna, David Gratzer, Damminda Alahakoon, Mary Jane Esplen

**Affiliations:** 1 de Souza Institute University Health Network Toronto, ON Canada; 2 Department of Psychiatry University of Toronto Toronto, ON Canada; 3 College of Professional Studies Northeastern University Toronto, ON Canada; 4 Faculty of Medicine University of Toronto Toronto, ON Canada; 5 The Michael G DeGroote School of Medicine McMaster University Hamilton, ON Canada; 6 Research Centre for Data Analytics and Cognition LaTrobe University Melbourne Australia; 7 Department of Computer Science University of Toronto Toronto, ON Canada; 8 The Department of Supportive Care Princess Margaret Cancer Centre Toronto, ON Canada; 9 Faculty of Social Work University of Toronto Toronto, ON Canada; 10 Centre for Addiction and Mental Health Toronto, ON Canada

**Keywords:** artificial intelligence, natural language processing, online support groups, supportive care in cancer, recommender system

## Abstract

**Background:**

The negative psychosocial impacts of cancer diagnoses and treatments are well documented. Virtual care has become an essential mode of care delivery during the COVID-19 pandemic, and online support groups (OSGs) have been shown to improve accessibility to psychosocial and supportive care. de Souza Institute offers CancerChatCanada, a therapist-led OSG service where sessions are monitored by an artificial intelligence–based co-facilitator (AICF). The AICF is equipped with a recommender system that uses natural language processing to tailor online resources to patients according to their psychosocial needs.

**Objective:**

We aimed to outline the development protocol and evaluate the AICF on its precision and recall in recommending resources to cancer OSG members.

**Methods:**

Human input informed the design and evaluation of the AICF on its ability to (1) appropriately identify keywords indicating a psychosocial concern and (2) recommend the most appropriate online resource to the OSG member expressing each concern. Three rounds of human evaluation and algorithm improvement were performed iteratively.

**Results:**

We evaluated 7190 outputs and achieved a precision of 0.797, a recall of 0.981, and an F1 score of 0.880 by the third round of evaluation. Resources were recommended to 48 patients, and 25 (52%) accessed at least one resource. Of those who accessed the resources, 19 (75%) found them useful.

**Conclusions:**

The preliminary findings suggest that the AICF can help provide tailored support for cancer OSG members with high precision, recall, and satisfaction. The AICF has undergone rigorous human evaluation, and the results provide much-needed evidence, while outlining potential strengths and weaknesses for future applications in supportive care.

## Introduction

Cancer and its treatment can significantly decrease the psychological well-being of patients and their families. Emotional distress, particularly related to symptoms of depression, is common among cancer patients and is associated with poor treatment adherence, reduced quality of life, and higher mortality rates [1-3]. The COVID-19 pandemic has amplified this psychological burden, resulting in a global rise in mental distress, especially among cancer patients, because of immunological concerns [4]. Virtual care, such as care from online support groups (OSGs), has become increasingly important in health care delivery, particularly with the more recent impact of COVID-19 that has resulted in the need for social distancing and minimal travel. OSGs offer a convenient and economical solution for those who cannot attend in-person support groups, and successfully reduce patient distress and anxiety [5-8].

Synchronized professionally led OSGs engage participants in therapeutic interactions. Group leaders facilitate the sharing of personal experiences among group members with similar challenges. The aim is to foster a mutually supportive environment to achieve an increased sense of empowerment via the vicarious learning that occurs through group membership and an increased sense of control through being better informed about the conditions [9].

A recent paradigm shift in health care, described as the learning health system, refers to a system of care involving the extraction of actionable information to inform clinical decisions whilst measuring patient experience responses for continued quality improvement [10]. Advances in artificial intelligence (AI), such as machine learning–based natural language processing (NLP), afford the development of learning systems that allow for real-time monitoring and responding to multiple participant care needs in virtual care settings. In a larger project, we leveraged machine learning–based NLP technology to monitor group session activities, track participant outcomes, detect psychosocial concerns in real time, and respond to these concerns automatically [11]. Our AI-based co-facilitator (AICF) system was developed to (1) identify participants at risk for increased emotional distress; (2) monitor in-session engagement and group cohesion levels, providing real-time alerts for the therapist; (3) generate postsession participant profiles that visualize individual emotional trajectories and psychosocial concerns; and (4) automatically suggest tailored online resources to participants based on their messages and participant profiles. Thus, the AICF personalizes support without adding burden to the patient or the therapist. Further, the application of medical resource recommender systems within the AICF can enhance individualized patient access to quality-verified resources that are tailored to the unique needs of patients. This study will report on the training process and performance of the AICF recommender system.

There are numerous applications of AI systems for health care delivery, including treatment recommendations, health education, and symptom management for patient populations [12-15]. A medical information search engine and recommender system called *personal health information recommender* (PHIR) provides personalized information based on individual patient profiles [13]. PHIR has a knowledge base of 855 online resources, which are registered by experts in the cancer domain. This knowledge base allows PHIR to tailor resources based on the user’s medical conditions and user ratings on the resource selection history, and to perform similarity matching. PHIR incorporated qualitative feedback from physicians and patients to improve its performance and has shown promising results. Additionally, Vik [12] is a conversational agent equipped with intent classification and entity recognition to provide personalized text messages in response to common questions about medical conditions. The results of a blinded randomized controlled study of 142 breast cancer patients demonstrated noninferiority in user-rated quality between answers provided by Vik and those provided by a physician [12].

Although these AI applications were rated by users before deployment, the actual outputs of these recommender systems have seldom undergone rigorous testing or evaluation by human medical experts. More studies are needed to demonstrate the efficacy of health care recommender systems, particularly for supportive care in cancer [16].

## Methods

### Platform and Training Data Set

de Souza Institute offers CancerChatCanada (CCC) that has national, professionally led, synchronous, and text-based OSGs for cancer patients and caregivers in collaboration with 6 provincial agencies in Canada. OSGs vary in length, aims, and group intervention models. All groups are manually based and consist of 6 to 8 sessions. Patient participants were recruited through CCC as well as the webpage and social media accounts (Facebook and Twitter) of de Souza Institute. Patients had to be diagnosed with cancer and able to speak English to be included. The exclusion criterion was the presence of distress needing immediate psychological care. Group sessions built on each other, with each session focusing on a specific theme. In sessions, therapists facilitate discussions based on weekly readings, address concerns, attend to the emotional needs of the members as they emerge, and employ group therapeutic factors that promote a continuous sense of mutual support among 6 to 10 members [17]. The OSG typically employs self-management skills that can empower participants as suggested by the Chronic Care Model [18]. The model posits that through empowering patients with self-management knowledge and resources, patients will become informed and engaged as active participants of care, contributing a collaborative partnership with the health care team toward improved outcomes [18]. OSG therapists routinely recommend additional information postsession that provides education around diagnoses and various coping practices, such as mindfulness and positive psychology interventions, to enhance self-management support. To date, CCC therapists have curated about 37 online resources and webpages that cover a variety of topics on cancer self-management, including physical and psychological symptoms, diagnostics, and treatment options (surgery, hormonal therapy, biotherapy, and chemotherapy), as well as caregiving issues, such as loss and bereavement, for all cancer patients. Additionally, there is a body of information on advanced or metastatic diseases and their diagnosis; management of symptoms, such as pain, constipation, diarrhea, anxiety, and depression; end-of-life discussions, such as advanced care planning; concerns faced by young people; and lifestyle guides on food safety and exercise. To enhance the virtual care system, we designed the AICF to identify psychosocial concerns and automatically suggest the most relevant online resources based on in-session conversations.

### Ethics Approval

This study has been approved by the University Health Network Research Ethics Board (CAPCR Study ID 18-5354). Participants provided informed consent before signing up for the OSG.

### NLP-Based AICF Algorithm

**Figure 1:**
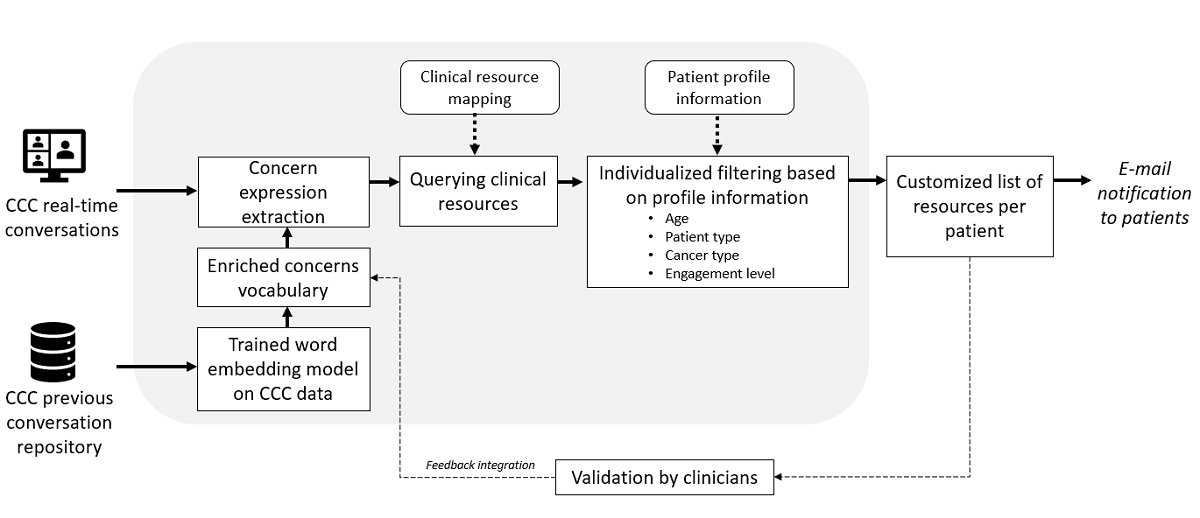
Overview of the artificial intelligence–based co-facilitator recommender system framework. CCC: CancerChatCanada.

**Figure 2:**
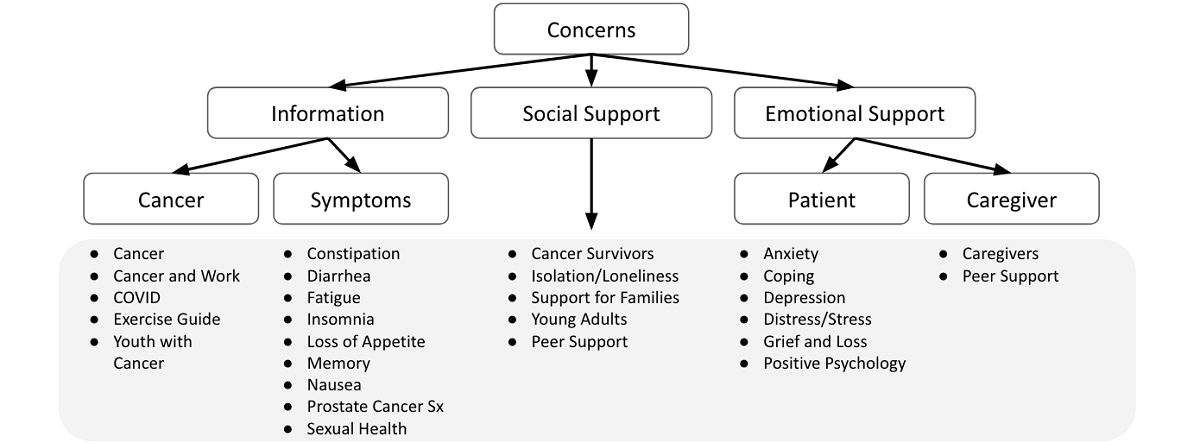
Taxonomy of the common psychosocial challenges of cancer patients. Patient concerns identified by the artificial intelligence–based co-facilitator were scored by a team of medical students and clinical experts based on a taxonomy created using their domain expertise. Sx: symptoms.

The AICF [11] was developed using an NLP-based approach with customization capabilities (Figure 1). First, a corpus of CCC chat sessions (approximately 80,000 messages) was used to train the AICF using word2vec, a word embedding model [19]. This model enabled creating a vector representation for each word in the corpus, thus positioning semantically similar expressions in closer proximity. Second, a team of therapists provided a list of common psychosocial concern keywords (Figure 2) that were fed into the trained word2vec model as inputs to generate semantically similar expressions by participants in session transcripts. Next, we queried for semantically similar expressions in the annotated sample. This enriched vocabulary list was used to extract concerns expressed in conversations. This allowed capturing of the terms and phrases related to each concern from patient posts. Third, once the concerns were identified, a concern-response matrix was used to match the best-suited resources for the patient. Finally, individual attributes were used to score the list of clinical resources to create the most appropriate recommendations. These attributes included age, cancer type, patient type (eg, caregiver status), symptoms of depression and anxiety, and engagement level in the group. This resulted in highly customized recommendations that best suited each patient (Figure 1).

### Psychosocial Concern Identification and Resource Database Evaluation

The team developed a literature-based list of psychological concerns relevant to cancer patients (Multimedia Appendix 1) and organized them into a taxonomy that formed the basis for AICF resource mapping (Figure 2) [20,21].

We reviewed 37 online resources curated by CCC therapists. Each resource was evaluated on a set of parameters adapted from the SQuaRE-Aligned Portal Data Quality Model (SPDQM), a model for website content quality evaluation [22,23]. This method aligns with the International Organization for Standardization (ISO) standard for software and data quality [24]. The quality parameters used are as follows: (1) accessibility, (2) understandability, (3) relevancy, (4) validity, and (5) attractiveness and readability (Multimedia Appendix 2).

Each online resource was rated on a Likert scale from 1 to 3 for the parameters listed above (1, *poor quality:* the resource should not be recommended; 2, *moderate quality:* the resource should be recommended to the patients with specific concerns or requests; 3, *high quality:* the resource would be recommended).

Only resources of moderate to high quality were included in the final list of resources available for AICF’s recommendation, and included resources were paired with the most appropriate psychosocial concerns (Table 1). Each resource was rated twice by 2 evaluators (BP and RH) who were blinded to each other’s rating. Consensus was reached through discussion with a third evaluator (YWL) to resolve discrepancies.

### AICF Performance Evaluation

The AICF was applied to the chat history of new OSGs, and outputs were scored by 2 medical students (BP and RH) using a confusion matrix. Recall, precision, and F1 score were used as evaluation metrics [25]. F1 score is the harmonic mean of precision and recall, which takes both false positives and false negatives into account to produce a single measure of performance.

Using the established concern domains (Table 1), the team assessed whether the AICF system (1) correctly identified each output instance (true positive), (2) incorrectly identified an output instance (false positive), (3) correctly identified the lack of an output (true negative), or (4) missed the concern in a statement (false negative). All false-positive and false-negative recommendations were analyzed for their underlying reasons and addressed to improve the AICF in subsequent rounds.

Given that the AICF was designed to read deidentified data sentence by sentence, the human raters were lenient regarding true-negative outputs that may have potentially indicated a concern, but the subject and context of how the concern applies remained ambiguous. The raters would rate “true negative” on the following phrase example: “Yes, my social worker tells me that all I can do is listen and be there for him. But that's really hard to do.” This phase may be interpreted as the struggles of a caregiver or a patient having difficulties coping; the role of the support group member and subject of concern remains ambiguous, and accurate resources cannot be recommended without additional context. Likewise, the rater rated “true negative” on the following phrase: “most don’t want to feel bad, and they can say very heartless things.” This phase may be interpreted as a support group member sharing personal feelings or as an observation that was used to connect and empathize with other members in the chat. Although flagging such ambiguous phrases may increase the sensitivity of the AICF, given that the objective of the AICF is to provide appropriate resources while avoiding information overload, we were lenient with negative outputs that had such ambiguity.

The evaluation results were used to retrain the model, while linguistic rules, part-of-speech tagging, and filtering based on the patient profile were applied to handle exceptions such as negations, past tense, and idioms of expression. Evaluators’ feedback using their domain expertise was used to improve AICF’s performance over the evaluation rounds until it achieved F1 >0.80 before deployment in real-time OSG sessions for beta testing [26].

Participants received an email containing the AICF-tailored recommendations postsession. Users evaluated AICF’s recommendations. Automatically generated emails asked the current participants to further evaluate the system. The users judged each recommended resource on usefulness by answering the following question: “Our system has recommended some resources for you based on the last chat session. Please let us know if the links are helpful or not by clicking on the *Useful* or *Not Useful* button below.” We also recorded the number of clicks on the recommended resources. Participant characteristics are presented in Table 2.

**Table 1:** The finalized concern-resource matrix.

Concerns	Type of resource							
	Website	Phone line	Learning modules	Online support group	PDF	Apps/games	Videos	Mindfulness
Newly diagnosed	Patient and Caregiver Cancer Connection Newly Diagnosed	Cancer support helpline	MyGrief.ca	Cancer Connection	—^a^	—	—	Nucare Manual
Anxiety & depression	Worried, Scared or Anxious Sadness and Depression	Cancer support helpline	MyGrief.ca	—	Sleeping Well Manual Anxiety Depression	—	—	Nucare Manual
Distress & intense emotions	Managing Stress	Cancer support helpline	MyGrief.ca	—	—	—	—	Nucare Manual
Grief & loss	Loss and Grief	Cancer support helpline	MyGrief.ca	—	—	—	Living My Culture	Nucare Manual
Isolation & loneliness	—	Cancer support helpline	—	Cancer Connection	—	—	—	Nucare Manual
COVID	COVID-19 and cancer	—	—	—	—	—	—	Nucare Manual
Finances & employment	Cancer and Work	—	—	—	Returning to Work	—	—	—
Caregiver support	—	Caregiver helpline	—	Cancer Support Community	—	—	—	—
Support for families	Family Support	—	—	—	—	Cancer in my family	—	—
Symptom management: Insomnia	Sleeping Well Manual	—	—	—	—	—	—	—
Symptom management: Pain	—	—	—	—	—	Pain and treatment side effects	—	—
Sexual health	—	—	—	—	Sexual Health	—	—	—
Symptom management: General	Symptom Management	—	—	—	—	—	—	—
Adolescents & young adults	Young Adult Cancer Cancer Fight Club	—	—	Young Adult Cancer	—	—	—	—

^a^Not available.

**Table 2:** Participant characteristics.

Characteristic		Value (N=48), n (%)
**Gender**		
	Female	43 (90)
	Male	4 (8)
	Unknown	1 (2)
**Age group (years)**		
	18-24	0 (0)
	25-34	3 (6)
	35-44	8 (17)
	45-54	10 (21)
	55-64	18 (37)
	65+	9 (19)
**Location**		
	British Columbia	18 (37)
	Ontario	14 (29)
	Alberta	7 (15)
	Other provinces	9 (19)
**Type of cancer**		
	Breast	24 (50)
	Gynecological	3 (6)
	Colorectal	5 (10)
	Head and neck	3 (6)
	Other cancers	12 (25)
	Unknown	1 (2)
**Treatment status**		
	Active treatment	8 (17)
	Posttreatment	22 (46)
	Other	18 (37)

## Results

A total of 35,600 outputs from the AICF on the CCC chat history were extracted over 3 evaluation rounds. The months of the data collected were February 2020, April 2020, and June 2020. A random sample of 20% unique statements with AICF’s decision outputs (n=7190) was evaluated by human raters using a confusion matrix. Example phrases from each category of the matrix are provided in Multimedia Appendix 1.

Among false negatives, the AICF failed to recognize culturally specific idioms of concern, which was reflected in the high number of errors. For example, the AICF failed to recognize the phrases “heart feels heavy” or “want to run away” as distress, “exhaustion” as fatigue, and “HER2” as breast cancer. Keywords in false-negative outputs were identified by human evaluators and used to retrain the AICF algorithm for improvement. As a result, the second and third rounds of evaluation added 75 and 17 new terms, respectively, to the AICF concern bank. This adjustment improved the false-negative rate from 54.8% (69/126) in the first round to 30.8% (16/52) in the second round and 6.9% (2/29) in the third round (Table 3).

**Table 3:** Classification accuracy.

Variable				Round 1 (N=4774), n		Round 2 (N=1195), n			Round 3 (N=1221), n
**Accuracy**				4648		1143			1192
	True positive		84			85		106	
	True negative		4564			1058		1086	
**Total inaccurate**				126		52			29
	False negative		69			16		2	
	**False positive**		57			36		27	
		Phrase ambiguity	28		9		5		
		Reference to future/past	19		7		3		
		Reference to others	4		10		13		
		Offering opinion	6		10		6		

False-positive outputs were classified into 1 of the following 4 subcategories: (1) *Phrase ambiguity:* there was insufficient information in the statement to fully assess whether a key concern was present; (2) *Reference to the future or past:* the statement maker was sharing a possible future or past event with other group members; (3) *Reference to others:* the statement maker refers to a concern pertaining to a person other than themselves; (4) *Offering an opinion:* the statement maker is offering their personal opinion or experience regarding a concern mentioned by another group member.

False positives were addressed by additional tagging techniques tailored for each underlying reason. The details are elaborated in the Discussion. Although the rate of false positives increased over 3 evaluation rounds (Table 3), this was most likely due to an increased sensitivity of the recommender system arising from the expanded vocabulary that was applied to address the false negatives, and the net result of these adjustments was an improvement in the F1 score from 0.571 in round 1 to 0.766 in round 2 and 0.880 in round 3 (Table 4).

**Figure 3:**
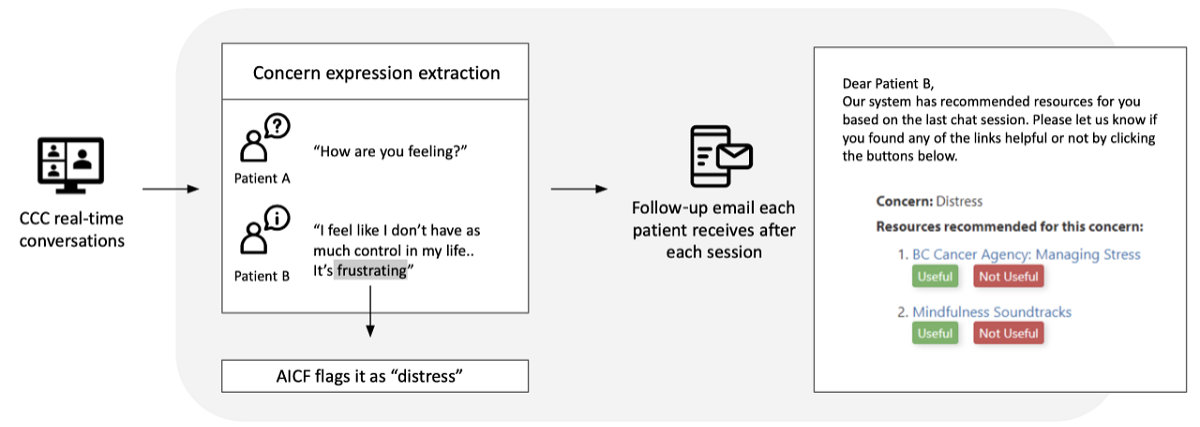
Patient experience with the artificial intelligence–based co-facilitator (AICF) recommender system. CCC: CancerChatCanada.

Figure 3 illustrates the patient experiences with the AICF recommender system. The recommender system was tested in a convenient sample of 5 OSGs, reaching 48 participants. Each participant was recommended an average of 11.3 unique resources, ranging from 2 to 40. Twenty-five (52%) of these participants clicked at least one of the recommended resources. These 25 participants viewed an average of 4.4 (39.1%) tailored resources. Among the participants who viewed resources, 19 (76%) rated them as “useful” (Figure 3).

**Table 4:** Precision, recall, and F1 score following each round of artificial intelligence–based co-facilitator evaluation.

Round	Precision	Recall	F1 score
1	0.596	0.549	0.571
2	0.702	0.842	0.766
3	0.797	0.981	0.880

## Discussion

### Principal Findings

This study evaluated AICF’s performance in identifying concerns and recommending resources for cancer patients based on transcripts from OSGs. The large amount of available information online can be overwhelming for resource seekers, especially for those who are affected by cancer. The aim of the AICF is to recommend high-quality resources that are tailored to concerns identified on the basis of each patient’s OSG chat history. A recommendation system based on patients’ needs expressed in the group discussion can potentially reduce the burden on patients to find the correct information and the burden on online therapists who need to respond to multiple participants simultaneously. The preliminary results show that the initial performance was low, indicated by an F1 score of 0.571, although accuracy was high (97.4%). For subsequent evaluation rounds, the AICF was retrained on the basis of feedback from human evaluators, which improved the performance to an F1 score of 0.880 by the third round of evaluation. These results demonstrate that the AICF displays sufficient accuracy in identifying concerns expressed by OSG participants and recommending relevant resources that can help to increase tangible support and service quality without incurring increased workload for therapists. Nineteen (76%) patients who viewed the AICF-recommended resources found them useful.

The AICF is a one-of-a-kind recommender system running behind the scenes of an OSG service without imposing on the therapist or participants. To date, there have been very few studies adopting a human expert in their system validation process. Compared to previous recommender systems, such as PHIR and Vik [27], the AICF adopted a human evaluator feedback loop and exhibited high performance and enhanced personalized support. The AICF performed a robust human evaluation on over 7000 outputs to produce values for accuracy, recall, and F1 scores. The AICF is unique in that it aims to provide automatic detection of psychosocial concerns and delivery of tailored resources for self-management. This technology augments therapist-led OSG sessions, while the other systems relied on patients actively seeking resources and using a search engine for resource delivery.

Among the recommender systems designed for health care delivery, the AICF is highly comparable to a conversational agent, Vik [27], based on common medical questions and physician answers. Vik uses intent classification and entity recognition to process user input texts. Intent classification identifies keywords from the user’s textual inputs and classifies them into one of the predetermined question categories. Entity detection identifies names or titles in the user’s inputs and classifies them into predefined categories. However, the AICF is embedded in an OSG and uses a statistical and rule-based approach with word embeddings, in which a subset of relevant keywords is extracted as intent, serving as inputs to the recommender. Furthermore, the AICF differs from Vik in that we incorporated user profile information, such as type of cancer, age, engagement level, anxiety and depression symptomatology, and caregiver status, to produce highly tailored recommendations. Another major difference is the fact that Vik was trained with a database consisting of questions asked by the users to their health professionals, while the AICF was trained on chat history data consisting of human-to-human text-based conversations in OSG format. The training data allowed the AICF to understand more diverse psychosocial concerns, but they are more complex to process.

The AICF showed high accuracy (97.4%) in the initial assessment, and it was stable over evaluation rounds. False negatives were reduced by expanding AICF’s vocabulary bank to include key terms that had been missed, resulting in a greater than 8-fold reduction in the false-negative rate between round 1 and round 3. However, continuous monitoring and retraining by feedback from human raters will be required for the AICF to be sensitive to idiom use in different contexts and scenarios. Future work should explore the use of a language model [28] to detect the idiomatic and metaphorical parts in sentences.

For false positives, the AICF identified concerns that were deemed incorrect by the human evaluators. These were categorized into the following 4 subcategories: (1) phrase ambiguity, (2) reference to the future or past, (3) reference to others, and (4) offering an opinion.

#### Phrase Ambiguity

There was insufficient information in the statement to fully assess whether a key concern was present. The text was primarily characterized by short messages in which contextual information was missing. One or more keywords of psychosocial concern were present and were picked up by the AICF in the absence of contextual information. Phrase ambiguity was complicated by using a deidentified data set. All identifiable personal health information, including user handle names, hospital names, and doctor names, had been removed in accordance with the guidelines from the research ethics board. This often leads to disjointed data in which phrases are fragmented. Currently, the AICF is unable to link conversations between specific participants, resulting in lost information regarding who is replying to whom or which statements the speaker agreed or disagreed with. We also removed the therapist’s statements to minimize the contamination of group outcomes arising from the therapist’s validation of the group discussion. This resulted in the loss of contextual information for the AICF, contributing to the number of false positives identified. Future work should look into different deidentification methods that can better protect the linkage of conversations without compromising participant confidentiality.

#### Reference to the Future or Past

The statement maker was sharing his/her past experiences or future events that had not yet happened. For example, participants shared an experience, triggering the AICF to identify a concern and recommend resources. However, the phrases indicated to the raters that the participant was no longer actively dealing with the identified concern. To address these themes in false-positive outputs, a speech tagging technique was added to the algorithm after the second round of validation to detect the use of past and future tenses. Future work will explore other word embedding models, such as sense2vec, to improve performance further.

#### Reference to Others

The AICF identified concerns and recommended resources to participants when participants were in fact referencing the stories of a third party. The addition of a linguistic rule to detect story-telling, such as the use of third person pronouns, may help improve false-positive outputs. Once more chat transcripts become available, it will be a feasible adjustment to further improve the precision of the AICF.

#### Offering an Opinion

The statement maker was offering his/her personal opinion or experience regarding a concern mentioned by another group member. Future studies can explore modeling the relationship between messages to recognize the conversation thread.

### Limitations

Apart from the limitations identified above, which are common as AI continues to improve itself, the 37 curated resources included in our recommender system can be seen as a relatively small set of information support resources in cancer care. These resources were selected for their relevance to assist OSG participants in dealing with the psychosocial challenges of living with different cancers. However, such resources could also be seen as too generic by participants and insufficient to meet their needs for a specific cancer. This may partially explain the fact that only 52% of the participants accessed a recommended resource. Additionally, the resources included were rated by medical professionals; however, there is obvious merit to additional evaluation by a more neutral party whose health literacy is more representative of the general public and patients who would utilize the AICF. The patient population included in this study was also likely more technologically savvy compared to the general public given that they had to be competent in maneuvering online webpages and social media to sign up for the OSG. While this may not be representative of all cancer patients, with the continued rise of internet usage and the strong need for additional remote support options with the COVID-19 pandemic, we predict that the population this system is geared toward will continue to become more representative of the general cancer patient population over the years. Next steps will involve conducting focus groups with OSG participants to explore their opinions on the AICF and expanding the resource rating team to include diverse backgrounds and perspectives in the rating process. Future work will expand the psychosocial resources to include those for particular cancers and develop NLP to recognize specific cancer types. Future work should also assess ethnicity/cultural parameters related to the AICF system.

### Conclusion

Owing to increased mental health care demands and barriers for accessing in-person care, virtual care has become paramount in the provision of supportive care. We have embedded the AICF within OSGs to increase personalized support and expand patient self-management capacities by recommending credible online resources. All these goals can be achieved without additional work from therapists. Future projects include user focus groups, development of cancer-specific recommender systems, expansion to additional languages, and ultimately randomized controlled trials to inform effectiveness and ensure further development of policies, such as mandating AI-enhanced OSGs as the first line of patient education to build self-management capacities for cancer and chronic diseases. Eventually, this line of research will inform our health system on the use of AI for future personalized supportive care delivery.

## Appendix

Multimedia Appendix 1 Themes, keywords, and examples of artificial intelligence–based co-facilitator outputs.

Multimedia Appendix 2 Resource evaluation tool.

## Abbreviations

AI: artificial intelligence
AICF: artificial intelligence–based co-facilitator
CCC: CancerChatCanada
NLP: natural language processing
OSG: online support group
PHIR: personal health information recommender
