# Supplementary material for: Providing Care Beyond Therapy Sessions With a Natural Language Processing–Based Recommender System That Identifies Cancer Patients Who Experience Psychosocial Challenges and Provides Self-care Support: Pilot Study
Source: JMIR Cancer. 2022 Jul 29;8(3):e35893. doi: 10.2196/35893 (PMC9377447; doi:10.2196/35893)
Supplement: Multimedia Appendix 1 [file cancer_v8i3e35893_app1.pdf]

Multimedia Appendix 1: Themes, keywords, and examples of artificial intelligence–based co-facilitator outputs

True Positive Themes	Examples
Anxiety	“anxiety has been a bigger issue for me than anything physical”
Cancer survivor	“I met with the ******** in the Legislature and he announced it in the Legislature and introduced me as a ** advocate and survivor. It was really quite emotional.”
Caregiver	“So, it's not just the demands of the physical caregiving, but also replacing the work that was done by the ill individual.”
Constipation	“Yes, constipation along with the bowel issues.”
Coping	“I did that several times - sometimes it works other times it's a struggle.”
Depression	“I joined a group therapy for depression and am waiting to see a therapist”
Distress	“My mother has a habit of calling me, hearing my voice and then exclaiming how well I sound. I find it incredibly frustrating. She has no interest in finding out how I'm actually doing. A lot of time she does that I've actually been super unwell and tired.”
Exercise Guide	“I am trying to get more exercise”
Fatigue	“I am being treated for depression and anxiety but can't deal with the side effects from the chemo....the fatigue,the soreness and I am ********* I am getting *********'s as I am so forgetful......”
Grief and Loss	“I think grief is part of this illness. I ****** for the person I was before cancer. I can't go back to being that person ever again.”
Insomnia	“My insomnia keeps rearing its ugly head again and it is hard to manage sometimes when I am traveling as well.”
Memory	“Right , the memory, I forgot. Actually getting panic attacks because of it”
Nausea	“I just started another oral chemo. It makes me a little nauseous but otherwise not bad.”
Prostate Cancer	“I believe we had the same surgery about the same time. ... I

	am having some incontinence and ongoing bowel problems. Not sure if it is from the surgery or the radiation . Feel like my belly has dropped”
**False Positive Themes**	**Examples**
Not enough information given (ambiguity)	“its a neat exercise”
Reference to a past or future event	Once I found out what it was I stopped associating the fatigue ... with the cancer chemo etc...
Reference to other group members or third party	“the General Manager is a Breast Cancer survivor”
Offering opinion to the group	“I agree that expressing and dealing with fears is a good approach.”
**False Negative Themes**	**Examples**
Caregiver	“is there a chat line for care givers?”
Coping	“well i feel totally useless since I had this cancer”
Distress	“I'm overwhelmed”
Fatigue	“*** too weak to go out anymore, and cant stay awake long enough for people to visit”
Finance	“It is. I feel like I have to choose between quality of life or paying bills.”
Insomnia	“I am truly looking forward to being able to fall asleep and stay asleep. It has been years and my mind and body need the rest”
