# Supplementary material for: Providing Care Beyond Therapy Sessions With a Natural Language Processing–Based Recommender System That Identifies Cancer Patients Who Experience Psychosocial Challenges and Provides Self-care Support: Pilot Study
Source: JMIR Cancer. 2022 Jul 29;8(3):e35893. doi: 10.2196/35893 (PMC9377447; doi:10.2196/35893)
Supplement: Multimedia Appendix 2 [file cancer_v8i3e35893_app2.pdf]

Multimedia Appendix 2: Resource Evaluation Tool

Link	Name of the resource	Who is the target population?	What is the name of the organization?	Which platform do they use? (ie. online, phone line, in-person)	Which formats are resources in? (ie. readings/resources, videos, phone call, online chat, personal/group Tx)	Is there any geographical restrictions?	Brief comments about the resource	Quality Rating	Concern(s) that we'd recommend for/updated links
https://sunnybrook.ca/content/?page=pynk-education-body-image-breast-cancer	Body Image Breast Cancer	Breast Cancer patients struggling with body image issues	Sunnybrook Hospital	Online	Readings	N	Easy readings	3: easy reading	Breast Cancer (body image)
https://www.virtualhospice.ca/en_US/Main+Site+Navigation/Home.aspx	Canadian Virtual Hospice	Cancer patients, family members, health care professions seeking information of palliative care	Canadian Virtual Hospice	Online	Videos/Readings/Print Materials	N	Resources on various topics surrounding palliative care - e.g. emotional/ spiritual health, decisions, financial benefit information; has ask a professional feature to ask doctors/nurses/social workers/pharmacists about specific health concerns (response via email within 3 days); also has information for professionals	2: overall site is easy to navigate, but the link is not specific to the concern	Coping, Greif and Loss
https://www.canceranddwork.ca/	Cancer and Work	Cancer survivors wanting/planning to return to work	McGill University, BC Cancer Agency, & de Souza institute	Online	Readings, self-assessment guides	N	Detailed resources for the entire process of returning to work about cancer treatment - initial self-assessment, workplace accommodations, disability benefits/insurance	3: thorough, user friendly, and specific	Cancer and work

https://cancerconnection.ca/home	Cancer Connection	Cancer patients requiring connections and social support	Canadian Cancer Society	Online	Discussion forum	N	Conversation among patients and volunteers who share similar experience via comments on forums	3: easy to navigate, good for patients who are looking for the community with people who are going through the same thing	Coping
https://cancerfightclub.com/	Cancer Fight Club	Young adult cancer patients seeking information, resources, community	Jewish General Hospital; Hope & Cope	Online	Regular live webinars, readings	N - but Montreal based	Has regularly scheduled zoom webinars for yoga, meditation, art, cooking etc., readings/resources for dealing with cancer as a young adult; previously offered in-person groups/retreat in Montreal but assuming that's put on hold due to COVID-19	2: recommend for patients looking for workshops, not as user friendly as some other sites and less readings/resources	Young adult survivors
http://www.cancerinmyfamily.ca/	Cancer in my Family	For children dealing with cancer in their family	BC Cancer Agency	Online	Interactive online activities ("games")	N	Kid friend interface with several online activities they can explore that provide education about cancer and encourage them to identify their feelings	3: looks like a fun, well-made interactive platform for kids	Support for families<br>Young children
http://www.cancerindex.org/clinks5c.htm	Cancer Index	Cancer patients and families requiring further info	Cacner Index Organization	Online	Stats and resources	N	Canada Cancer stats, and links to government organizations or advocate groups within Canada	1: too broad; hard to navigate	N/A
https://www.cancersupportcommunity.org	Cancer Support Community	Cancer patients requiring further info and support	Cancer Support Community	Phone/Online	readings/resources, Helpline, online live chat	N/US	Information on cancer types and treatments, links to cancer support communities (mainly in US) , also offer helpline and online live chats	2: user friendly, but the links to in-person communities are only availble for US locations, and the community is also mailny US-based.	Coping, Isolation
https://www.cancersupportcommunity.org/	Cancer Support Community	Cancer patients requiring information and support	Cancer Support Community	Online/Phone	Readings/videos, discussion forum, live chat, helpline	N	Helpine M-F 9-9PM, S-S 9-5PM; online "mylifeline" platform where patients can document their journey for family/friends, and access discussion forums with other patients	3: easy to navigate, but in-person facilites are located in US	Depression, Coping, Distress: (specific link to their helpline) https://www.cancersupportcommunity.org/cancer-support-helpline<br><br>For patients looking for in-person programs and facilities in Canada, use this link: https://csl.cancer.ca/en<br><br>For patients looking for an online community in Canada, use this link: https://cancerconnection.ca/home#-featured-content-

https://www.cdc.gov/cancer/survivors/index.htm	Cancer Survivorship Resources	Cancer survivors, caregivers/family members of cancer survivors	CDC - Centers for Disease Control and Prevention	Online	Readings, Videos	N	General guides/readings for staying healthy, regaining normalcy etc. after cancer treatment; inspirational stories/videos from other cancer survivors	3: user friendly, covers wide breadth of topics related to life after cancer, but update the link so that it directs patients to a more specific/relevant area for survivors	Cancer Survivors
https://www.partnershipagainstcancer.ca/browse-topics/	Cancer View	Cancer patients and families requiring further info	CPAC (Canadian Partnership against Cancer)	Online	Readings/resources	N	Database; Can search for different types of cancer, type of care, risk factors, etc.	1: not suitable for general population; hard to navigate and obtain any information	N/A
http://www.bccancer.bc.ca/health-info/coping-with-cancer	Coping with Cancer	Cancer patients seeking treatment, espeically who are in BC	BC Cancer	Online	Readings/resources	N/BC	Information on cancer and treatments can be useful for everyone, but mainly useful for patients and families looking for specific centers, treatment options, and support groups or group therapy programs that are offered in BC.	2: overall site is easy to navigate, but the link is not specific to the concern	Grief and Loss: http://www.bccancer.bc.ca/health-info/coping-with-cancer/emotional-support/loss-and-grief<br>Anxiety: http://www.bccancer.bc.ca/health-info/coping-with-cancer/emotional-support/worried-scared-or-anxious<br>Managing Stress: http://www.bccancer.bc.ca/health-info/coping-with-cancer/emotional-support/managing-stress<br>Sadness and Depression: http://www.bccancer.bc.ca/health-info/coping-with-cancer/emotional-support/sadness-and-depression<br>Exercise Guide: http://www.bccancer.bc.ca/health-info/coping-with-cancer/exercise-support#Recommendations<br>Support for Families: http://www.bccancer.bc.ca/health-info/coping-with-cancer/talking-to-my-family<br><br>Symtoms:<br>Constipation: http://www.bccancer.bc.ca/health-info/coping-with-cancer/managing-symptoms-side-effects/constipation-(caused-by-opioid-pain-medications)
http://shared.desouzainstitute.com/cancerchat/Resources/COVID-19AndCancer-PatientInfo.pdf	COVID-19 and cancer	Cancer patients worrying about COVID	Ontario health	Online	Reading	N	Typical COVID-19 documentation	2: informative, but no emotional support or further resources available	COVID<br><br>Cancer and COVID: https://www.cancer.ca/en/support-and-services/support-services/cancer-and-covid19/?region=on provides more in-depth and user friendly information<br>Coping with cancer during COVID: https://www.cancer.ca/en/support-and-services/support-services/coping-with-cancer-during-the-covid19/?region=on<br>Talking to an informative specialist: https://www.cancer.ca/en/support-and-services/support-services/talk-to-an-information-specialist/?region=on"
http://www.gr	Group Loop	Teenagers with cancer or who's	Not sure - affiliated with	Online	Readings	N	Short reads/easy to navigate site on	3: short, easy to read, good for youth/teens	Teens with cancer

ouploop.org/		dealing with a loved one having cancer	Cancer Support Community?				various topics for teens (eg. school, visiting the doctor, dealing with parents)		
https://www.cancercareontario.ca/en/symptom-management	Guides for patients: How to Manage Your Symptoms	Cancer patients seeking information on symptom managements	Cancer Ontario	Online	Readings/resources	N	Guidelines on symptoms such as: anxiety, bone health, bowel function or sexual activity (prostate cancer), constipation/diarrhea, delirium, depression, dyspnea, fatigue, fever, and hand-foot syndrome. Also offer exercise guidelines and tracking sheets	3: user friendly, but the link can get even more specific when necessary	Anxiety: https://www.cancercareontario.ca/en/symptom-management<br>Bone Health: https://www.cancercareontario.ca/en/symptom-management/35071<br>Bowl Function - **Prostate Cancer:** https://www.cancercareontario.ca/en/symptom-management/35041<br>Constipation: https://www.cancercareontario.ca/en/symptom-management/3146<br>Delirium: https://www.cancercareontario.ca/en/symptom-management/3136<br>Depression: https://www.cancercareontario.ca/en/symptom-management/3986<br>Diarrhea: https://www.cancercareontario.ca/en/symptom-management/3151<br>Dyspnea: https://www.cancercareontario.ca/en/symptom-management/3126<br>Exercise: https://www.cancercareontario.ca/en/symptom-management/53116<br>Fatigue: https://www.cancercareontario.ca/en/symptom-management/3991<br>Fever: https://www.cancercareontario.ca/en/symptom-management/4001<br>Hand-foot Syndrome: https://www.cancercareontario.ca/en/symptom-management/35066<br>Hormonal Symtpoms - **Prostate Cancer:** https://www.cancercareontario.ca/en/symptom-management/35046<br>Loss of Appetite: https://www.cancercareontario.ca/en/symptom-management/3141<br>Low Platelet Count: https://www.cancercareontario.ca/en/symptom-management/35076<br>Nausea and Vomiting: https://www.cancercareontario.ca/en/symptom-management/3131<br>Neutropenia: https://www.cancercareontario.ca/en/symptom-management/35081<br>Oral Care: https://www.cancercareontario.ca/en/symptom-management/3156<br>Pain: https://www.cancercareontario.ca/en/symptom-

									management/3121<br>Sexual Health:<br>https://www.cancercareontario.ca/en/symptom-management/35051<br>Sleep Disturbance:<br>https://www.cancercareontario.ca/en/symptom-management/3996<br>Incontinence - **Prostate Cancer:**<br>https://www.cancercareontario.ca/en/symptom-management/35056<br>Urinary Problem - **Prostate Cancer:**<br>https://www.cancercareontario.ca/en/symptom-management/35061
https://knightscabin.com/	Knights Cabin	Cancer survivors and their supporters seeking retreat/building of community to move forward	Knight's Cabin	In-person/online	In-person weekend retreat; online webinars	Retreat in Kamloops, BC	2 night/3 days retreat for cancer survivors and caregivers; $580 for cancer patients and extra $100 for guest/caregiver; next one scheduled for November 2020 (contingent on COVID-19 status); also offers regular free online live webinars/Q&A on various topics (eg finances and cancer, exercise with cancer)	1-2: unique opportunity (weekend retreat); but very specific - for patients looking for a retreat/can travel to BC/afford the cost	Cancer survivors (only if they can afford/access/are looking for this type of support community)
https://livingmyculture.ca/culture/first-nations/	Living My Culture	Care providers (health care professionals, counsellors, family members/caregivers)	Canadian Virtual Hospice	Online	Videos/Readings/Print Materials	N	Videos of people from 11 different cultures - First Nations, Inuit, Metis, Chinese, Ethiopian, Filipino, Indian, Iranian, Italian, Pakistani, Somalian - about their perspectives/values/traditions when dealing with severe illness, healing, grief	2: culture specific concerns/videos/support. Only recommend for individuals who identify as one of the 11 cultures listed	Support for families:<br>https://livingmyculture.ca/topic/care-for-the-patient-and-family/<br>Grief and loss: https://livingmyculture.ca/topic/after-death-and-ceremonies/<br><br>**Only recommend for individuals who identify as one of the 11 cultures listed**
https://planwellguide.com	Miscellaneous Resources	Patients seeking help for sleep problem or stress management		Online	Resources	N	Links to self-help guildelines for sleep problems, relaxation recordings, mindfulness exercise, and useful apps for stress management	2: recommend for patients who are looking for mindfulness techniques, apps to help with stress, guided relaxation; but is not an informative/educational resource	Anxiety, Distress, Coping
http://www.mygrief.ca/	MyGrief	Those dealing with grief from losing loved ones	Canadian Virtual Hospice	Online	Interactive online modules	Canada ($25 fee for out of country)	Online modules for members to work through on their own pace - topics include "what am I feeling, talking to children/families,	3: specific website for grief/loss, has thorough modules that patients can work through for various topics/concerns	Distress, Coping, Grief and loss, Depression

							asking for help"; also offers feature where you can ask a professional and a member of the Canadian Virtual Hospice Team will respond in three days; discussion forums available		
https://www.nccn.org/patients/	National Comprehensive Cancer Network	Cancer patients and families requiring further info	National Comprehensive Cancer Network	Online	Readings	N	Guidelines for patients on cancer treatments, featuring Qs to ask doctors, patient-friendly illustrations and explanations.	2: each guideline provides a thorough overview of each type of cancer, but the link is not specific to the concern	Cancer Info:<br>Bladder Cancer:<br>https://www.nccn.org/patients/guidelines/cancers.aspx#bladder<br>Breast Cancer:<br>https://www.nccn.org/patients/guidelines/cancers.aspx<br>Colon Cancer:<br>https://www.nccn.org/patients/guidelines/cancers.aspx#colon<br>Kidney Cancer:<br>https://www.nccn.org/patients/guidelines/cancers.aspx#kidney<br>Leukemias and Lymphomas:<br>https://www.nccn.org/patients/guidelines/cancers.aspx#leukemiasAndLymphomas<br>Liver Cancer:<br>https://www.nccn.org/patients/guidelines/cancers.aspx#hpLiver<br>Lung Cancer:<br>https://www.nccn.org/patients/guidelines/cancers.aspx#lung<br>Ovarian Cancer:<br>https://www.nccn.org/patients/guidelines/cancers.aspx#ovarian<br>Pancreatic Cancer:<br>https://www.nccn.org/patients/guidelines/cancers.aspx#pancreatic<br>Prostate Cancer:<br>https://www.nccn.org/patients/guidelines/cancers.aspx#prostate<br>Rectal Cancer:<br>https://www.nccn.org/patients/guidelines/cancers.aspx#rectal<br>Skin Cancer:<br>https://www.nccn.org/patients/guidelines/cancers.aspx#squamous<br>Thyroid Cancer:<br>https://www.nccn.org/patients/guidelines/cancers.aspx#thyroid<br>Uterine Cancer:<br>https://www.nccn.org/patients/guidelines/cancers.aspx#uterine
https://support.desouzainstitu	Nucare Manual	Patients attempting to go back to their normal routine	Jewish General Hospital and McGill Hospital	Online	Reading and worksheets	N	Worksheets on: Mindfulness, cognitive reframing, communication skills,	2: easy to follow, patients can pace themselves. It may get boring however, as it is not as interactive	Destress, Coping

te.com/kb/article/54-nucare-manual							problem solving, social support, healthy lifestyle, and goal settings		
https://ontariocaregiver.ca/find-support/helpline/	Ontario Caregiver Helpline	Caregivers of cancer patients who need support/have questions	Ontario Caregiver Organization	Phone/Online	Helpline, online livechat, readings	N - but more tailored to Ontario	24/7 helpline for caregivers, online livechat M-F 7AM-9PM; can provide information for support groups, respite care in the area, financial assistance etc, English and French speakers, resources/readings also available on the site	3: user friendly	Support for families
https://docs.google.com/document/d/1-hZBURI-0iGRN9z50XNYVIcuIlAd3-A5zElreOA5caM/edit	Plan Well	Patients hoping to prepare an advanced care planning for a serious illness in the future	Dr. Daren Heyland	Online	Readings and a questionnaire	N	A questionnaire allows users to identify their preferences on 1. Quality of remaining life vs. Prolonging lifetime, and 2. Receiving resuscitation and ICU care. This can then be printed or sent to their doctors via email.	3: user friendly	Coping, Grief and loss
https://rethinkbreastcancer.com/	Rethink Breast Cancer	Young breast cancer patients	Rethink Breast Cancer	Online	online Facebook group, readings	N	Readings/stories for young women dealing with breast cancer, can request access to join the Rethink Young Women's Network which is a Facebook group for young women with breast cancer	2: good resource but specifically for young women diagnosed with breast cancer	Breast cancer support<br>Newly diagnosed<br>Young cancer survivors/Young patients
https://support.desouzainstitute.com/kb/article/55-sleeping-well-manual	Sleeping Well Manual	Cancer patients with insomina	Dr.Sheila Garland	Online	Reading and worksheets, resources (books)	N	5 Wks Cognitive-behavioral therapy manual describing self-management steps for chronic insomnia: daily sleep diary, setting goals for sleep efficiency (sleeping/time in bed), applying sleep restriction and stimulus control,	3: user friendly, thorough long-term guided support	Insomnia

							practicing imaginary relaxation technique and cognitive reframing, and finally creating a plan for dealing with relapse		
https://www.youngadultcancer.ca	Young adult Cancer Canada	Young adult cancer patients seeking community	Young Adult Cancer Canada (YACC)	Online/in-person	Reading/resources, online Facebook group, In-person events	N	Access to private fb group, local based in-person social meetings in 8 cities across Canada, 4 days retreat, survivor conference, as well as articles and stories for young patients	3: user friendly, online community available, personal stories	Young adult survivors
